# Identifying the Most Autonomy-Supportive Message Frame in Digital Health Communication: A 2x2 Between-Subjects Experiment

**DOI:** 10.2196/14074

**Published:** 2019-10-30

**Authors:** Eline Suzanne Smit, Chamoetal Zeidler, Ken Resnicow, Hein de Vries

**Affiliations:** 1 Department of Communication Science Amsterdam School of Communication Research University of Amsterdam Amsterdam Netherlands; 2 School of Public Health University of Michigan Ann Arbor, MI United States; 3 Department of Health Promotion Care and Public Health Research Institute Maastricht University Maastricht Netherlands

**Keywords:** health communication, health behavior, personal autonomy, internet, health promotion, healthy diet, self-determination theory

## Abstract

**Background:**

The effectiveness of digital health communication may be increased by enhancing autonomy supportiveness.

**Objective:**

This study aimed to identify the most autonomy-supportive message frame within an intervention for increasing vegetable intake by testing the effect of the following 2 strategies: (1) using autonomy-supportive language and (2) providing choice.

**Methods:**

A Web-based 2 (autonomy-supportive vs controlling language)×2 (choice vs no choice) experiment was conducted among 526 participants, recruited via a research panel. The main outcome measures were perceived autonomy support (measured using the Virtual Care Climate Questionnaire, answered with scores 1 to 5), perceived relevance (measured with one question, answered with scores 1 to 5), and overall evaluation of the intervention (measured with 1 open-ended question, answered with scores 1 to 10).

**Results:**

Choice had a significant positive effect on the overall evaluation of the intervention (*b*=.12; *P*=.003), whereas for participants with a high need for autonomy, there was a significant positive effect on perceived relevance (*b*=.13; *P*=.02). The positive effect of choice on perceived autonomy support approached significance (*b*=.07; *P*=.07). No significant effects on any of the three outcomes were observed for language.

**Conclusions:**

Results suggest that provision of choice rather than the use of autonomy-supportive language can be an easy-to-implement strategy to increase the effectiveness of digital forms of health communication, especially for people with a high need for autonomy.

## Introduction

### Digital Health Communication to Date

Digital forms of health communication, for example, Web-based computer-tailored interventions, can be a cost‑effective strategy for health promotion [1-3]. However, the effect sizes found in previous studies have so far remained small [1], which suggests room for improvement. As the public health impact of digital health communication can only be maximized when we use the immense reach of the internet and optimize its efficacy (impact=reach×efficacy [4]), testing strategies that might increase efficacy is a priority.

Such effect improvement may be achieved by moving beyond a focus on *what* health information is provided to a focus on *how* this information is provided. Until recently, health communication scholars have mainly focused on tailoring the content of digital health communication based on receivers’ current health behavior and behavioral determinants [5]. However, variations in the communication style (ie, the *how*) that is used to deliver health communication messages are believed to have different effects as well [6]. Previous studies indicate, for instance, that autonomy-supportive communication strategies may enhance the impact of face-to-face health communication interventions, such as counseling, by facilitating the internalization of motivation [7]. Yet, little is known about the effect of autonomy-supportive strategies in digital forms of health communication [8]. Therefore, this study aimed to identify the most autonomy-supportive message frame by testing the effects of the following 2 strategies intended to increase perceptions of autonomy support: (1) the use of autonomy-supportive language; and (2) the provision of choice, within a Web-based computer-tailored intervention aimed at increasing vegetable intake.

### Autonomy-Supportive Communication Styles

Previously, both autonomy-supportive and more directive communication styles have been identified and studied in offline forms of health communication [9,10]. According to Self-Determination Theory (SDT) [8,11], supporting autonomy is an important prerequisite for achieving autonomous motivation for health behavior change. Autonomous motivation has been found to be an important predictor of actual behavior change and subsequent positive health outcomes [7,12]. In the face-to-face setting, providing autonomy support involves strategies such as the elicitation and acknowledgment of a person’s perspectives, the provision of a clear rationale for change, supporting the person’s volition, and using autonomy-supportive or noncontrolling language and offering choice [13]. Such strategies have, however, not been studied to a large extent within the context of digital health communication. We found 2 digital health studies that manipulated autonomy support. In the first, the authors report positive effects of the provision of autonomy support by a virtual health care provider. However, the authors do not provide much detail regarding how the support was provided [11]. The second study elaborately describes the operationalization of autonomy support provided by a computer-based personal trainer using choice (eg, “choose whichever one works better for you” vs “do this”), the acknowledgement of feelings (eg, “some people feel intimidated and those feelings are normal” vs “some people feel intimidated, but that’s not useful”), and minimal evaluations or judgments (ie, providing recommendations vs telling participants what they should do), although without reporting on the results of the study [8]. A recent meta-analysis of techniques to promote motivation for health behavior change even distinguished 18 different techniques aimed at the promotion of need satisfaction and autonomous motivation, the provision of choice and the use of noncontrolling language being 2 of them [14]. We are aware of only 1 digital health study that focused on the effects of different autonomy-supportive strategies in virtual care settings without virtual health care providers involved. At the same time, most Web-based computer-tailored health communication interventions do not involve a virtual health care provider, but rather provide tailored advice with the program as a main source. In that study [15], the authors report no differential effects of an autonomy-supportive versus controlling tone when used in Web-based computer-tailored alcohol reduction messages on perceived autonomy support from and reactance toward these messages, neither did they find a moderation effect for baseline need for autonomy. However, it should be noted that participants in the study, regardless of the message tone used, generally rated the messages positively, providing high levels of autonomy support. This suggests that there may not have been adequate conceptual separation between message conditions [15].

### Providing Autonomy Support in Web-Based Computer Tailoring

Computer-tailored health communication uses a computerized process to adjust message content based on the individual users’ personal characteristics (eg, behavior, personality, attitudes, and beliefs), with the goal of increasing perceptions of personal relevance [16]. As such, tailored health communication interventions have been found to be more successful in attracting and keeping the receiver’s attention [16,17] and in achieving effortful processing of information [18] when compared with nontailored interventions. When tailored content is provided using an autonomy-supportive communication style, similar to face-to-face autonomy-supportive communication, receivers ideally perceive the intervention as more supportive of their autonomy, and co-occurring feelings of personal freedom may rise. This increased sense of freedom might encourage the receiver to process in-depth only those parts of the intervention that are most appealing to himself or herself personally, thereby further increasing perceptions of personal relevance.

Given the positive effects found of an autonomy-supportive communication style (ie, including the use of autonomy-supportive language and the provision of choice) in the face-to-face setting [9,10] and virtual clinician context [8,11], in addition to theory and evidence on tailoring effects, we formulated the first 2 hypotheses.

- H1: The use of autonomy-supportive language will result in (a) higher perceived autonomy support, (b) higher perceived relevance, and (c) a more positive overall evaluation of the intervention compared with the use of controlling language.
- H2: The provision of choice will result in (a) higher perceived autonomy support, (b) higher perceived relevance, and (c) a more positive overall evaluation of the intervention compared with no provision of choice.

### The Need for Autonomy as a Moderator

Although SDT suggests a universal need for autonomy, there may be individual differences in how autonomy needs influence message impact [19]. Some people prefer to choose their own path toward lifestyle improvement, whereas others prefer to be guided by clear-cut expert advice [20]. Although SDT scholars have recognized these individual differences [7,12], only few have taken these differences into account when developing health communication strategies, and none have done so in the context of digital health communication. Not taking into account individual differences in the need for autonomy may result in communicating digital health information that does not fit people’s personal needs, making it less likely to be read and less likely to be considered as personally relevant [21]. As a consequence, messages are less likely to be centrally processed, reducing behavioral impact. This idea finds support in findings from 2 studies that investigated the effects of printed health communication aimed to increase colorectal cancer screening [20] and fruit and vegetable intake [22]. Both studies found that for people with greater need for autonomy—operationalized as a preference for autonomy-supportive communication compared with directive communication—newsletters that were communicated in an autonomy-supportive tone were more effective in changing target behaviors than newsletters that were communicated in a more directive tone.

On the basis of these previous studies, we propose that in the context of digital health communication, baseline need for autonomy will interact with the intervention manipulations as follows:

- H3: The effects of the use of autonomy-supportive language (vs the use of controlling language) on (a) perceived autonomy support, (b) perceived relevance, and (c) the overall evaluation of the intervention will be stronger for respondents with a high need for autonomy than for respondents with a low need for autonomy.
- H4: The effects of the provision of choice (vs no provision of choice) on (a) perceived autonomy support, (b) perceived relevance, and (c) the overall evaluation of the intervention will be stronger for respondents with a high need for autonomy than for respondents with a low need for autonomy.

## Methods

### Design and Participants

To test the hypotheses, an experiment with a 2 (language use: autonomy-supportive vs controlling language)×2 (choice: provided vs not provided) between-subjects design was conducted within the context of an existing Web-based computer-tailored intervention module aimed at increasing vegetable consumption [23]. Participants were recruited via *PanelClix*, an International Organization for Standardization–certified research panel [24].

A total of 728 Dutch adult participants started the experiment. Participants who did not give their informed consent (1/728, 0.1%) were not interested in eating—or continuing to eat—250 g of vegetables per day or did not provide an answer to this inclusion question (7/728, 1.0%) were excluded and not randomized. Of 720 randomized participants, 604 (83.8%) completed the entire intervention and questionnaire. However, we excluded participants with problematic or implausible response patterns: 7/604 (0.9%) participants took too long to fill in the questionnaire (3 SDs or more above the mean completion time), 23/604 (3.0%) participants filled in the questionnaire too fast (ie, <5 min), 2/604 (0.3%) participants filled in 0 as their weight, 9/604 (13.0%) participants had an extremely high vegetable consumption (3 SDs or more above the mean vegetable consumption), and 46/604 (6.0%) participants did not answer the 7 process evaluation questions in a logically consistent manner (ie, they filled in the same response for all questions even when items were scaled in opposing directions). For some of the participants, more than one of these problems were encountered; a total of 79/604 (13.1%) participants were excluded, and the final sample consisted of 525 participants. Of them, 231/525 (44.0%) were men, and 294/525 (56.0%) were women, with age ranging from 18 to 65 years (mean 43.35 years, SD 13.80). About half of the participants (261/525, 49.7%) were highly educated, and participants consumed on an average 183.80 g of vegetables daily (SD 96.45). Their average body mass index (BMI) was 25.52 kg/m^2^ (SD 4.95).

A Consolidated Standards of Reporting Trials flow diagram is provided in Multimedia Appendix 1, whereas Table 1 provides an overview of the final sample’s characteristics.

**Table 1:** Sample characteristics (N=525).

Variables		Value, n (%)	Value, mean (SD)	Range
**Sex**				
	Male	231 (44.0)	—^a^	—
	Female	294 (56.0)	—	—
Age (years)		—	43.35 (13.80)	18-65
Length (cm)		—	174.64 (9.69)	154-200
Weight (kg)		—	78.07 (17.63)	30-192
Body mass index (kg/m^2^)		—	25.52 (4.95)	12.49-52.08
**Education**				
	Low	41 (7.8)	—	—
	Middle	222 (42.3)	—	—
	High	261 (49.7)	—	—
	Other	1 (0.1)	—	—
Vegetable consumption		—	183.80 (96.45)	0-700

^a^Not applicable.

### Procedure

This study was approved by the ethics committee of the University of Amsterdam (reference number: 2016-PC-7205). First, participants received a brief explanation about the study aims and procedures as well as information about their rights and the confidential handling of their data. After participants provided their Web-based informed consent, they were asked about their intention to (continue) eating 250 g of vegetables per day, that is, the Dutch guideline for vegetable consumption [25]. When participants had a positive intention (yes vs no), they were randomly assigned to 1 of the following 4 conditions: autonomy-supportive language, or controlling language, with provision of choice, or without provision of choice (see Table 2 for the number of participants per condition). Subsequently, participants were asked about their demographics. After completion of the intervention (which is described in more detail in the following section), participants reported their perceived autonomy support and personal as well as their overall evaluation of the intervention. The postintervention questionnaire also queried participants’ need for autonomy. The average time that participants needed to complete the questionnaire was approximately 15 min (to be precise: 964.69 seconds; SD 981.74). Participants were rewarded by means of 150 Clix, the incentive of PanelClix, worth approximately €1.88.

**Table 2:** Experimental conditions.

Choice	Language use, n (%)		Total, n (%)
	Autonomy supportive	Controlling	
Yes	147 (28.0)	124 (26.6)	271 (51.6)
No	141 (26.9)	113 (21.5)	254 (48.4)
Total	288 (54.9)	237 (45.1)	525 (100.00)

### The Web-Based Computer-Tailored Intervention

The Web-based computer-tailored intervention was based on an intervention previously developed by Schulz et al, which aimed to improve several lifestyle-associated behaviors (ie, physical activity, vegetable consumption, fruit consumption, alcohol intake, and smoking cessation) and was called myHealthyBehaviour. As the study into the effectiveness of myHealthyBehaviour showed that the percentage of noncompliance with Dutch health guidelines was highest for vegetable intake (ie, 68%) [23], this study specifically focused on the vegetable consumption module of the intervention. For this study, we therefore changed the intervention’s name to MyVegetableConsumption. In addition, we incorporated the updated Dutch guideline of consuming a minimum of 250 g rather than 200 g of vegetables daily [25].

The intervention consisted of 4 steps, each consisting of a set of questions and tailored feedback based on their answers, following an initial assessment of respondents’ vegetable consumption. The first step looked at the advantages and disadvantages participants experienced with regard to consuming enough vegetables, for example, the (expensive) price of vegetables and their positive effect on one’s health. The second step looked at the influence of the social environment of the participants, including one’s partner, family, friends, and colleagues. The third step assisted participants in making preparatory plans to consume enough vegetables, for example, by bringing vegetables to work as part of their lunch. The fourth step looked at participants’ self-efficacy and aided them in making plans to cope with potentially difficult situations, for example, in busy times.

### The Manipulations

The tailored feedback participants received was written in either an autonomy-supportive or controlling language, and choice options were either provided or not provided throughout the intervention.

#### Language

Language was manipulated into autonomy-supportive language or controlling language that was used throughout the intervention, that is, in (the introductions to) the questions and the tailored feedback. Autonomy-supportive language was intended to provide participants a sense of volition over their decisions and used more tentative advice, for example, “you could try to bring snack vegetables to work.” In contrast, controlling language was more directive and definitive, for example, “you must bring snack vegetables to work!” [9,26].

#### Provision of Choice

Provision of choice was manipulated by either providing or not providing participants the possibility to choose if they wanted to make each out of 5 suggested preparatory plans (step 3) and to choose whether or not they wanted to make coping plans for each of the 7 potentially difficult situations described (step 4). Accordingly, participants who were provided with choice received feedback that was tailored based on the plans they chose (not) to make. Participants who were not provided with choice received 1 (nontailored) advice statement for preparatory planning and 1 (nontailored) advice statement addressing plans to cope with potentially difficult situations.

#### Pilot Test

A pilot test of the manipulations was conducted among experts in digital health communication and health message framing (N=8) and target group members (N=8), the latter varying in age, sex, and socioeconomic status. Experts were identified through the professional network of the first author, and target group members were identified through the first and second authors’ private networks. Both were invited to complete the intervention and accompanying evaluation questions and were asked to identify any ambiguities in the questions and/or feedback. Moreover, they were asked to indicate whether the intervention felt autonomy supportive or controlling and whether they experienced choice or not. On the basis of the results from the pilot test, several improvements were made to messages and assessments.

An example of a feedback message in autonomy-supportive and controlling language, combined with and without choice, is provided in Multimedia Appendix 2.

### Variables and Measures

#### Demographic and Other Background Variables

Several demographic variables were assessed, that is, sex, age, educational level, and marital status. BMI was estimated based on the participant’s self-reported height and weight, and weekly vegetable consumption was measured using a 4-item food frequency questionnaire [27].

#### Perceived Autonomy Support

Perceived autonomy support was the first dependent variable and was measured using the Virtual Care Climate Questionnaire [28]. The 15 items (eg, “I feel that MyVegetableConsumption has provided me with choices and options”) could be answered from 1 (*completely disagree*) to 5 (*completely agree*) and were transformed into a mean score (alpha=.96; mean 3.76, SD 0.77).

#### Perceived Relevance

Perceived relevance was the second dependent variable and was measured with 1 question (“I perceived the feedback messages as personally relevant”) that could be answered from 1 (*completely disagree*) to 5 (*completely agree*; mean 3.74, SD 1.02).

#### Overall Evaluation of the Intervention

Overall evaluation of the intervention was the third dependent variable and was measured by an open-ended question to give an overall grade for the intervention: “Please evaluate the intervention with a school grade from 1 to 10” (1=lowest grade and 10=highest grade; mean 7.50, SD 1.20).

#### Need for Autonomy

Need for autonomy, the main moderator variable, was measured with a 9-item scale. First, 6 items from the Health Causality Orientation Scale (HCOS) were included. The HCOS was developed by the third author based on the General Causality Orientation Scale [12] and looks at 4 causality orientations individuals may have when it concerns their health, namely, autonomous, controlled (experts), controlled (peers), and impersonal. To assess these orientations, participants were presented with 2 vignettes, in which they had to imagine that (1) they were discussing with a health professional how best to obtain their health-related goals and (2) they wanted to change their health behavior. Each vignette was followed by 4 items representing participants’ autonomous, controlled (experts), controlled (peers), and impersonal orientations, on a scale from 1 (*very unlikely*) to 5 (*very likely*). For this study, only the 6 items measuring participants’ autonomous and controlled orientation were considered, as the 2 items measuring impersonal orientations were not considered relevant for this study’s purposes. Second, 3 items were included based on previous measures for assessing communication style preferences [20,22], for example, “When it comes to my health, I would like to have an expert tell me what to do” (1=*completely disagree* and 5=*completely agree*). A factor analysis with all 9 items revealed 2 factors: (1) a factor with 3 items, representing the need for autonomy (alpha=.61; mean 4.16, SD 0.66) and (2) a factor with 6 items, representing the need for external control (alpha=.76; mean 2.94, SD 0.77).

### Data Analysis

First, we checked whether there was an equal distribution of demographic and other background variables across the conditions by conducting Chi-square tests and analyses of variance. If baseline differences were detected, correlations between these variables and the dependent variables were calculated. Variables that were not equally distributed across conditions and were correlated significantly with one or more of the dependent variables were included in subsequent analyses as covariates.

Second, regression analyses were conducted to test the effects of language and choice on each of the 3 dependent variables (ie, perceived autonomy support, perceived relevance, and overall evaluation of the intervention). When there appeared to be a significant interaction effect between (either of) the 2 conditions and (one of) the moderator(s), the interaction was dismantled by conducting a median split of the moderator and comparing outcomes between the 2 groups.

## Results

### Correlations

Sex (χ^2^_3_=6.5 *P*=.09), educational level (χ^2^_6_=10.8; *P*=.10), marital status (χ^2^_12_=19.1; *P*=.09), age (*F*_3,521_=0.54; *P*=.65), BMI (*F*_3,521_=0.63; *P*=.59), and vegetable consumption (*F*_3,521_=1.30; *P*=.27) were all distributed equally across the 4 conditions. As all variables were equally distributed across conditions, correlations between these variables and the dependent variables were not reported, and none of these variables were included in subsequent analyses as covariates. Mean scores including their SDs for each of the 3 dependent variables are, however, reported per condition in Table 3.

**Table 3:** Means (SDs) for dependent variables per condition (N=525).

Variables	Autonomy-supportive language and choice (n=147)	Autonomy-supportive language and no choice (n=141)	Controlling language and choice (n=124)	Controlling language and no choice (n=113)
Perceived autonomy support	3.77 (0.76)	3.70 (0.84)	3.82 (0.73)	3.76 (0.72)
Perceived relevance	3.77 (0.94)	3.67 (1.12)	3.80 (1.00)	3.69 (1.03)
Overall evaluation	7.60 (1.05)	7.33 (1.40)	7.66 (1.07)	7.42 (1.23)

### Hypothesis Testing

#### Perceived Autonomy Support

The positive effect of choice on perceived autonomy support approached significance (*b*=.07; *P*=.07), providing partial support for hypothesis 2a. For language, no significant main effect was found on perceived autonomy support. Neither were any interaction effects found between choice and language and the need for autonomy or need for external control. Therefore, hypotheses 1a, 3a, and 4a should be rejected. There were, however, significant main effects of the need for autonomy (*b*=.32; *P*<.001) and the need for external control (*b*=.32; *P*<.001) on perceived autonomy support. This means that participants with a high need for autonomy and participants with a high need for external control reported high levels of perceived autonomy support, independent of the message received. Table 4 provides the results of this analysis.

**Table 4:** Effect of choice, language use, and need for autonomy on perceived autonomy support.

Variables	*Beta (B)*	SE (*B)*	*Beta (b)*	*t test (df)*	*P* value	95% CI
Choice	.06	0.03	.07	1.85 (11,513)	.07	0.85 to 1.73
Language	−.03	0.03	−.04	−0.97 (11,513)	.33	−0.09 to 0.03
Need for autonomy	.37	0.05	.32	7.95 (11,513)	<.001	0.28 to 0.46
Need for external control	.32	0.04	.32	8.04 (11,513)	<.001	0.24 to 0.39
Choice×language	−.01	0.03	−.01	−0.30 (11,513)	.76	−0.07 to 0.05
Choice×need for autonomy	−.03	0.03	−.04	−1.03 (11,513)	.30	−0.09 to 0.03
Choice×need for external control	−.03	0.03	−.04	−0.96 (11,513)	.34	−0.09 to 0.03
Language×need for autonomy	−.03	0.03	−.04	−1.09(11,513)	.28	−0.09 to 0.03
Language×need for external control	−.01	0.03	−.01	−0.15 (11,513)	.88	−0.07 to 0.06
Choice×language×need for autonomy	.01	0.03	.02	0.40 (11,513)	.69	−0.05 to 0.07
Choice×language×need for external control	−.02	0.03	−.02	−0.55 (11,513)	.58	−0.08 to 0.04

#### Perceived Relevance

There was a significant interaction effect between choice and the need for autonomy on perceived relevance (*b*=.08; *P*=.04; data not reported). Therefore, we report outcomes separately for participants with a high need (ie, with a score <4.32) and participants with a low need for autonomy (ie, with a score >4.32), based on a median split procedure. This showed that for participants with a low need for autonomy, there was no effect of choice on perceived relevance, whereas for participants with a high need for autonomy, there was a significant positive effect of choice on perceived relevance (*b*=.13; *P*=.02). Although hypotheses 1b, 2b, and 3b need to be rejected, the results thus confirm hypothesis 4b. For participants with a high and a low need for autonomy, there was a significant main effect of the need for external control on perceived relevance, implying that participants with a high need for external control reported high positive levels of perceived relevance than respondents with a low need for external control. Table 5 provides full details of the results.

**Table 5:** Effect of choice, language use, and need for autonomy on perceived relevance for participants with a high and a low need for autonomy.

Variables		*Beta (B)*	SE *B*	*Beta (b)*	*t test (df)*	*P* value	95% CI
**Low need for autonomy (n=242)**							
	Choice	−.05	0.05	−.05	−0.86 (7,235)	.39	−0.15 to .06
	Language use	−.08	0.05	−.09	−1.40 (7,235)	.16	−0.18 to 0.03
	Need for external control	.36	0.08	.28	4.58 (7,235)	<.001	0.21 to 0.52
	Choice×language use	.01	0.05	.01	0.15 (7,235)	.89	−0.10 to 0.11
	Choice×need for external control	.06	0.06	.06	1.00 (7,235)	.32	−0.06 to 0.18
	Language×need for external control	−.12	0.06	−.12	−1.89 (7,235)	.06	−0.24 to 0.01
	Choice×language×need for external control	−.09	0.06	−.09	−1.50 (7,235)	.13	−0.22 to 0.03
**High need for autonomy (n=281)**							
	Choice	.15	0.06	.13	2.40 (7,274)	.02	1.74 to 2.59
	Language use	.02	0.06	.02	0.34 (7,274)	.73	−0.10 to 0.14
	Need for external control	.58	0.07	.44	8.20 (7,274)	<.001	0.44 to 0.72
	Choice×language use	−.02	0.06	−.01	−0.25 (7,274)	.80	−0.14 to 0.10
	Choice×need for external control	−.07	0.06	−.07	1.32 (7,274)	.19	−0.18 to 0.04
	Language×need for external control	−.02	0.06	−.02	−0.38 (7,274)	.70	−0.13 to 0.09
	Choice×language×need for external control	−.04	0.06	−.04	−0.68 (7,274)	.50	−0.15 to 0.07

#### Overall Evaluation

In terms of overall intervention rating, there was neither a significant interaction effect, rejecting hypotheses 3c and 4c, nor a significant main effect of language, rejecting hypothesis 1c. There was, however, a significant positive main effect of choice on the overall evaluation of the intervention (*b*=.12; *P*=.003). More specifically, the provision of choice was associated with a higher overall evaluation by the participants than no provision of choice, which confirms hypothesis 2c. Moreover, there were again main effects of the need for autonomy (*b*=.15; *P*<.001) and the need for external control (*b*=.32; *P*<.001), indicating that participants with a high need for autonomy and participants with a high need for external control evaluated the intervention significantly higher. Table 6 provides complete results of this analysis.

**Table 6:** Effect of choice, language use, and need for autonomy on overall evaluation of the intervention.

Variables	*Beta (B)*	SE *B*	*Beta (b)*	*t test (df)*	*P* value	95% CI
Choice	.14	0.05	.12	2.95 (11,513)	.003	0.05 to 0.24
Language use	−.04	0.05	−.03	−0.74 (11,513)	.46	−0.13 to 0.06
Need for autonomy	.27	0.08	.15	3.55 (11,513)	<.001	0.12 to 0.42
Need for external control	.49	0.06	.32	7.66 (11,513)	<.001	0.36 to 0.61
Choice×language use	−.00	0.05	−.00	−0.09 (11,513)	.93	−0.10 to 0.09
Choice×need for autonomy	.01	0.05	.01	−0.17 (11,513)	.87	−0.09 to 0.11
Choice×need for external control	−.07	0.05	−.06	−1.46 (11,513)	.15	−0.17 to 0.03
Language×need for autonomy	−.01	0.05	−.01	−0.24 (11,513)	.81	−0.11 to 0.09
Language×need for external control	.06	0.05	.05	1.22 (11,513)	.23	−0.04 to 0.16
Choice×language×need for autonomy	.06	0.05	.05	1.21 (11,513)	.23	−0.04 to 0.16
Choice×language×need for external control	−.07	0.05	−.06	−1.33 (11,513)	.19	−0.16 to 0.03

In a sensitivity analysis, we checked whether including a relative score of the need for autonomy (ie, score of need for autonomy–score of need for external control) as a potentially moderating variable, instead of 2 separate, unyoked variables for the need for autonomy and need for external control, yielded a change in results. The results were similar, yet 1 minor difference was observed; the marginally significant effect of choice on more perceived autonomy-support turned nonsignificant (data not reported).

## Discussion

### Discussion of the Results

This study aimed to identify the most autonomy-supportive message frame within a Web-based computer-tailored intervention for increasing vegetable consumption. To this end, based on prior empirical research and theory, we investigated the effects of 2 strategies, that is, using autonomy-supportive language and offering choice, among Dutch adults on 3 outcomes (ie, perceived autonomy support, perceived relevance, and the overall evaluation of the intervention). Moreover, we examined whether individual differences in the need for autonomy and need for external control moderated these effects.

First of all, there appeared to be a main effect of choice on the overall evaluation of the intervention compared with no provision of choice, as well as positive effects of choice on perceived autonomy support that approached significance. This is in line with the expectations we had based on previous studies conducted in the face-to-face setting [9] and on the persuasive effects of choice more generally [29]. This can be considered preliminary evidence that the provision of choice could be an effective strategy to increase the effectiveness of Web-based computer-tailored health communication.

With regard to the potentially moderating role of need for autonomy, a significant interaction effect with choice was found for the dependent variable of perceived relevance: only for participants with a high need for autonomy was there a significant positive effect of choice. This is in line with our expectations, as we hypothesized that the positive effects of the provision of choice would be stronger for respondents with a high need for autonomy and strengthens the idea that message frame tailoring based on the need for autonomy might be a promising avenue to advance digital forms of health communication [6]. This idea is also empirically supported by findings from a recent study by the first author and colleagues, showing that customization—the ability to self-tailor the mediated environment [30,31]—in mobile health apps leads to higher intentions to engage in physical activity for those with a greater need for autonomy, but not for those with a smaller need for autonomy.

On the other hand, the language manipulation resulted in neither significant main effects nor significant interaction effects with the needs for autonomy and external control were found for any of the 3 dependent variables. Thus, the tone used in our health communication messages did not impact our 3 outcomes. It appeared not to matter whether the participants received the Web-based computer-tailored intervention using an autonomy-supportive or controlling communication style. This is, surprisingly, in line with the results from a recently published study into the effects of autonomy-supportive versus controlling message frames on individual’s perceived autonomy support from and reactance toward such messages, studied in the context of a Web-based computer-tailored alcohol reduction intervention [15]. A potential explanation for this lack of effect—also mentioned by the authors from this recently published paper—might be derived from Politeness Theory [32]. In line with the operationalization we used in this study, Politeness Theory operationalizes autonomy-supportive language as not forceful language. In addition, however, this theory emphasizes the importance of perceived equality between the receiver and sender of the message. This implies that the receiver wants to be respected and seen as an equal by the sender of the message, or—in other words—their conversation partner, unless this conversation partner has a clear role of power over the receiver. If the conversation partner is perceived to be treating the receiver pedantically, although this is perceived to be outside of his or her power, the receiver might (un)consciously decide to resist the message and, consequently, not accept it—something also stressed by Psychological Reactance Theory [33]. As the source of the intervention was mentioned in the factsheet respondents received before entering the study and was intended to be perceived as expert (ie, 2 universities in combination with an innovative health consultancy agency), respondents in both conditions might have perceived the source of the intervention as having a clear role of power, resulting in the language manipulations no longer having any effect. To shed light on this, in future studies, it might be important to look at the moderating role of source characteristics and, for instance, investigate whether language and choice effects are different when the source of the message is (perceived as) an expert or a peer, as well as at resistance toward the message and/or source as a potential mediator of these effects.

### Implications and Suggestions for Future Research

The provision of choice resulted in the Web-based computer-tailored intervention being more positively evaluated and perceived as more autonomy supportive compared with no provision of choice, although the effect of perceived autonomy support only approached significance and should be interpreted with caution. For health communication practice, this may imply that the provision of choice could be an effective and easy-to-implement strategy to increase the effectiveness of digital forms of health communication. Given its potentially large reach, this increased effectiveness may improve the impact of this low-cost health behavior change strategy on public health. There is, however, still room for future research in this area as, in this study, the provision of choice was operationalized by providing participants the possibility to indicate to what extent they wanted to make several preparatory and coping plans that were recommended based on previous research findings. This may be interpreted as a combination of both verbal choice (in this case, emphasizing to the respondents that they could choose which of the recommended plans they wanted to make) and physical choice (in this case, physically giving respondents the opportunity to indicate their desire to make a certain plan or not). As it has previously been suggested that different types of choice may lead to different effects [29], future research efforts might aim to disentangle the effects of these different types of choice and of different operationalizations of verbal and physical choice, respectively.

Second, the interaction effect found between choice and respondents’ need for autonomy suggests that both health behavior change theorists and health communication professionals may need to take each individual’s communication style preferences (more) into account. Both SDT [7,12] and the I-Change Model [13] suggest the importance to tailor interventions to individual differences. SDT stresses the importance of need for autonomy in this regard, whereas the I-Change Model does not articulate which specific personal preferences need to be addressed as this is likely to depend on the topic, the target group, and the context. Consequently, the I-Change Model (ie, the theoretical framework used as a basis for the intervention studied) did not yet pay explicit attention to individual differences in the need for autonomy. The results from this study suggest that these sociocognitive models might actually benefit from doing so, suggesting an integration of theoretical concepts from different theories, a view also underlying the I-Change Model. For professionals, results suggest that when developing health communication interventions, people with a high need for autonomy—but not necessarily people with a low such need—might need to be provided with possibilities for choice throughout the intervention. In addition to the type of choice that was provided in this study (ie, the possibility to indicate to what extent participants wanted to make several preparatory and coping plans), other types of choice can be offered. For example, a recent study by the first author and colleagues shows that strategies such as customization and providing the respondent with explicit possibilities to choose could be used especially for people with a high need for autonomy. In contrast, system-driven tailoring strategies—tailoring based on an assessment of the individual respondent’s characteristics, with no explicit choice options provided—might be better suited for respondents with a low need for autonomy. It should be noted, however, that the interaction effect described was only found for one of the dependent variables, that is, perceived relevance, and that for people with a low need for autonomy, no *negative* effect of choice was found. As a consequence, results should be interpreted with caution, and further research into the moderating role of the need for autonomy—as well as the need for external control—is warranted.

### Strengths and Limitations

Some limitations also should be considered. First, we used perceived autonomy support, perceived relevance, and the overall evaluation of the intervention as outcome measures, assessed directly postintervention. Although we can assume that these measures would facilitate the internalization of motivation and ultimately predict health behavior change and its maintenance [7], future research efforts might want to consider measuring respondents’ motivation, intention to change, and/or actual behavior (change) using a longitudinal research design with a longer follow-up period. Second, we tested the effects of language and choice in the context of a Web-based computer-tailored intervention only. Although this was based on evidence from several previous studies [1] and we wanted to test the add-on effect of variations in message frame, future research might aim at testing whether the theoretical hypotheses also hold—may in fact, be stronger—in a nontailored context. Third, as participants in the choice conditions received feedback that was tailored based on the preparatory and coping plans they chose (not) to make and participants who were in the no-choice conditions received 1 (nontailored) advice statement for both preparatory planning and coping planning, it might be difficult to disentangle the effect of providing choice from the effect of an additional amount of content-tailored feedback. Future research could operationalize choice independently from the content tailoring, for example, by letting respondents choose whether and for which out of a predetermined set of potentially difficult situations they want to make a coping plan and comparing this with respondents instructed to make plans for all these situations—not providing them with any subsequent (content-tailored) feedback, or by letting respondents choose for a date at which they agree to start their lifestyle change, for example, to start eating more vegetables or to stop smoking versus providing respondents with a randomly generated start date. Finally, only respondents interested in eating—or continuing to eat—250 g of vegetables per day were included in the study. This inclusion criterion was deliberately chosen. As a positive motivation to change has been acknowledged by theory (eg, studies by de Vries et al [13] and Ajzen [34]) and evidence (eg, studies by Vangeli et al [35] and Smit et al [36]) to be a necessary prerequisite for actual behavior change, future research efforts might additionally explore the effects of language and especially choice among less motivated individuals.

### Conclusions

This study suggests that provision of choice rather than the use of autonomy-supportive language can be an easy-to-implement strategy to increase the effectiveness of digital health communication, especially for people with a high need for autonomy.

## Appendix

Multimedia Appendix 1 Consolidated Standards of Reporting Trials flow diagram.

Multimedia Appendix 2 An example of a feedback message in autonomy-supportive and controlling language combined with and without choice.

Multimedia Appendix 3 CONSORT-EHEALTH checklist (V 1.6.1).

## Abbreviations

BMI: body mass index
HCOS: Health Causality Orientation Scale
SDT: Self-Determination Theory
