# Supplementary material for: Identifying the Most Autonomy-Supportive Message Frame in Digital Health Communication: A 2x2 Between-Subjects Experiment
Source: J Med Internet Res. 2019 Oct 30;21(10):e14074. doi: 10.2196/14074 (PMC6914245; doi:10.2196/14074)
Supplement: Multimedia Appendix 1 [file jmir_v21i10e14074_app1.pdf]

Multimedia Appendix 1. CONSORT Flow Diagram

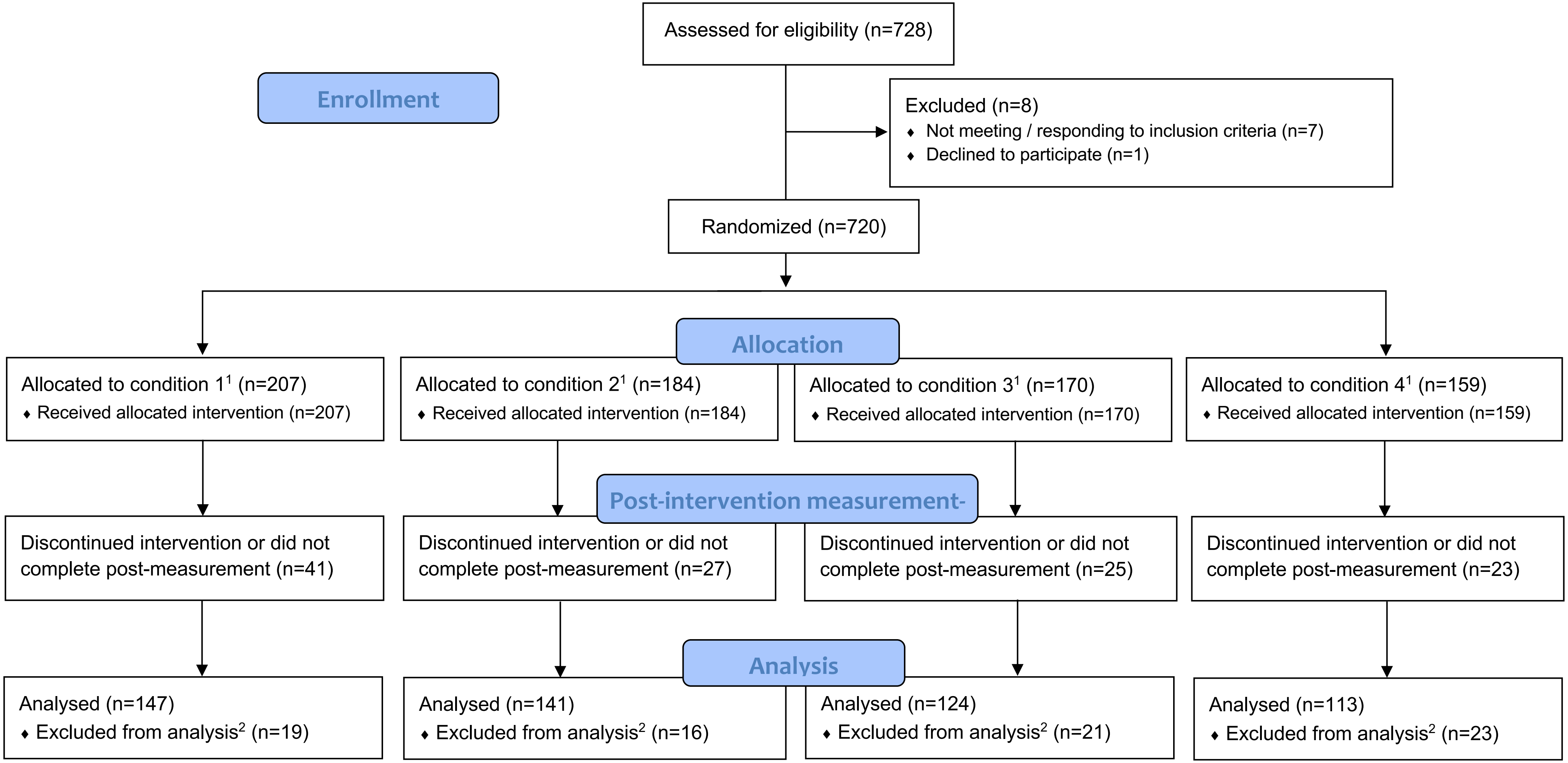

[1] Condition 1 = Autonomy-supportive language & choice; Condition 2 = Autonomy-supportive language & no choice; Condition 3 = Controlling language & choice; Condition 4 = Controlling language & no choice;

[2] Reasons for exclusion entailed not filling in the questionnaire seriously, taking too long to fill in the questionnaire, completing the questionnaire too fast, filling in zero as weight, having an extremely high vegetable consumption, and not answering the seven process evaluation questions seriously – or any combination of these reasons.
