# Supplementary material for: Identifying the Most Autonomy-Supportive Message Frame in Digital Health Communication: A 2x2 Between-Subjects Experiment
Source: J Med Internet Res. 2019 Oct 30;21(10):e14074. doi: 10.2196/14074 (PMC6914245; doi:10.2196/14074)
Supplement: Multimedia Appendix 2 [file jmir_v21i10e14074_app2.pdf]

Multimedia Appendix 2. An example of a feedback message in autonomy-supportive and controlling language combined with and without choice

	Choice	No choice
**Autonomy-supportive language**	<u>*Step 3. Your preparatory plans*</u><br><br>*If you are planning to eat more vegetables then you do at the moment, **it is important** that you think about how you will precisely do so. Research has shown that planning can help you to attain such goals. Below, you will find a couple of plans that you **could** make to prepare yourself well for eating more vegetables. From this list, you **can choose** for yourself those plans suit you best and will work best for you personally.*<br><br>*We do **like** to know which plans you will choose, however. Therefore, we **kindly ask** you to indicate the extent to which you choose for the plan to …*	<u>*Step 3. Your preparatory plans*</u><br><br>*If you are planning to eat more vegetables then you do at the moment, **it is important** that you think about how you will precisely do so. Research has shown that planning can help you to attain such goals. You **can**, for instance, inform the people in your environment about your good intention to eat more vegetables.*<br><br>*On the next page we will explain to you why you **could** make this plan if you want to attain your goal.*
**Controlling language**	<u>*Step 3. Your preparatory plans*</u><br><br>*If you are planning to eat more vegetables then you do at the moment, you **have to** think about how you will precisely do so. Research has shown that planning can help you to attain such goals. Below, you will find a couple of plans that you **should** make to prepare yourself well for eating more vegetables. From this list, **choose** for yourself those plans suit*	<u>*Step 3. Your preparatory plans*</u><br><br>*If you are planning to eat more vegetables then you do at the moment, you **have to** think about how you will precisely do so. Research has shown that planning can help you to attain such goals. You **must**, for instance, inform the people in your environment about your good intention to eat more vegetables.*

	you best and will work best for you personally.<br><br><br>*We do* ***want*** *to know which plans you will choose, however. Therefore,* ***indicate*** *the extent to which you choose for the plan to …*	*On the next page we will explain to you why you* ***should*** *make this plan if you want to attain your goal.*
